# Suspension syndrome: a potentially fatal vagally mediated circulatory collapse—an experimental randomized crossover trial

**DOI:** 10.1007/s00421-019-04126-5

**Published:** 2019-03-20

**Authors:** Simon Rauch, K. Schenk, G. Strapazzon, T. Dal Cappello, H. Gatterer, M. Palma, M. Erckert, L. Oberhuber, B. Bliemsrieder, H. Brugger, P. Paal

**Affiliations:** 1 Institute of Mountain Emergency Medicine, Eurac Research, Viale Druso 1, 39100 Bolzano, Italy; 2 Department of Sports Science, Medical Section, University of Innsbruck, 6020 Innsbruck, Austria; 3 Department of Anesthesiology, University Hospital LMU Munich, 80337 Munich, Germany; 4 Department of Sports Medicine, Pro Motus, 39100 Bolzano, Italy; 5 Department of Cardiology, F. Tappeiner Hospital, 39012 Merano, Italy; 6 Department of Internal Medicine I, Gastroenterology, Hepatology, Metabolism and Endocrinology, Medical University Innsbruck, 6020 Innsbruck, Austria; 7 Department of Anesthesiology, Garmisch-Partenkirchen Medical Center, 82467 Garmisch-Partenkirchen, Germany; 8 Department of Anesthesiology and Intensive Care Medicine, Brothers of St. John of God Hospital, Paracelsus Medical University, Kajetanerplatz 1, 5010 Salzburg, Austria

**Keywords:** Suspension syndrome, Harness hang syndrome, Suspension trauma, Climbing, Pathophysiology, Treatment

## Abstract

**Purpose:**

Suspension syndrome describes a potentially life-threatening event during passive suspension on a rope. The pathophysiological mechanism is not fully understood and optimal treatment unknown. We aimed to elucidate the pathophysiology and to give treatment recommendations.

**Methods:**

In this experimental, randomized crossover trial, 20 healthy volunteers were suspended in a sit harness for a maximum of 60 min, with and without prior climbing. Venous pooling was assessed by measuring the diameter of the superficial femoral vein (SFV), lower leg tissue oxygenation (StO_2_) and by determining localized bioelectrical impedance. Hemodynamic response was assessed by measuring heart rate, blood pressure, stroke volume, and left ventricular diameters. Signs and symptoms of pre-syncope were recorded.

**Results:**

Twelve (30%) out of 40 tests were prematurely terminated due to pre-syncopal symptoms (mean 44.7 min, minimum 13.4, maximum 59.7). SFV diameter increased, StO_2_ and the capacitive resistance of the cells decreased indicating venous pooling. Heart rate and blood pressure did not change in participants without pre-syncope. In contrast, in participants experiencing pre-syncope, heart rate and blood pressure dropped immediately before the event. All symptoms dissolved and values returned to normal within 5 min with participants in a supine position.

**Conclusions:**

Sudden pre-syncope during passive suspension in a harness was observed in 30% of the tests. Blood pools in the veins of the lower legs; however, a vagal mechanism finally leads to loss of consciousness. Time to pre-syncope is unpredictable and persons suspended on a rope should be rescued and put into a supine position as soon as possible.

**Electronic supplementary material:**

The online version of this article (10.1007/s00421-019-04126-5) contains supplementary material, which is available to authorized users.

## Introduction

Suspension syndrome (also called suspension trauma) describes a potentially life-threatening event induced by passive hanging on a rope or in a harness system in a vertical or near-vertical position (Flora 1972b; Pasquier et al. 2011; Roggla et al. 2008). Suspension syndrome does not only play a role in various occupational activities where harness systems are used, such as inspection, painting and construction on high-rise buildings, large facades, pylons, bridges, dams, off-shore platforms and power plants, but also in sports such as rock climbing, ice climbing, mountaineering, canyoning and caving (Pasquier et al. 2011). The first case series of suspension syndrome in climbers was reported in 1972: 10 out of 23 climbers who had fallen in the rope died after hanging in their harness, though they did not suffer any traumatic injuries (Flora 1972b). Since then, the pathophysiology of the suspension syndrome has been debated controversially (Lee and Porter 2007). The most widespread hypothesis assumes blood pooling in the lower limbs, prompting a reduction in cardiac preload and subsequently a decrease in cardiac output and tissue perfusion, eventually leading to loss of consciousness and cardiac arrest (Lee and Porter 2007). However, no study has ever proven this theory, and alternative pathophysiological mechanisms have been discussed (Halliwill et al. 2014; Roeggla et al. 1996). Specifically, a vagal mechanism leading to sudden bradycardia and hypotension has been hypothesized.

Since the first descriptions of suspension syndrome, the immediate aid by first responders has been debated (Thomassen et al. 2009). Some current recommendations still advise against placing a casualty in a supine position after being rescued from suspension and recommend a semi-recumbent position (Weems and Bishop 2003). The basis for this recommendation is the hypothesis that blood returning from the legs upon horizontal positioning would lead to an acute volume overload of the right heart and eventually cause rescue death. However, this hypothesis is based on expert opinion and case reports presented in 1972 (Flora 1972a) and has never been proven.

The main aim of our study was to elucidate the pathophysiological mechanism leading to suspension syndrome and to derive recommendations for prevention and treatment. In particular, we wanted to differentiate between a relevant reduction in cardiac preload due to venous pooling, and a vagally mediated event. In addition, we hypothesized that passive hanging in a harness with prior physical activity would increase the susceptibility and decrease the time to the occurrence of suspension syndrome, particularly if a vagal mechanism was involved. In fact, post-exercise syncope, although incompletely understood, is a known entity (Lowe and Petch 2000; O’Connor et al. 1999; Shen et al. 2017), and in real scenario, a certain amount of exercise usually precedes the fall into the rope.

## Methods

### Study approval and ethical background

This experimental randomized crossover study was approved by the Institutional Review Board of the General Hospital of Bolzano, Italy (no. 68-2015) and registered in ClinicalTrials.gov (trial number NCT02726776). The study was conducted according to the Declaration of Helsinki (World Medical Association Declaration of Helsinki: ethical principles for medical research involving human subjects 2013), Convention on Human Rights and Biomedicine (Convention for Protection of Human Rights and Dignity of the Human Being with Regard to the Application of Biology and Biomedicine: Convention of Human Rights and Biomedicine 1997) and the regulations of the Council for International Organizations of Medical Sciences (International ethical guidelines for biomedical research involving human subjects 2002). Written informed consent was obtained from all participants before enrolment.

### Study population

The study was performed on 20 healthy [American Society of Anesthesiologists (ASA) classification class 1], male, non-professional climbers (mean age 31.1, range 21–46 years).

Before enrolment, a medical history, physical examination and a 12-lead ECG were obtained and volunteers with pre-existing illnesses (ASA-class > 1) or any abnormal finding in the physical examination or the ECG were excluded. Participants did not take any medications and did not have a history of recurrent syncopal events. In an echocardiographic exam prior to the study, cardiopmyopathies or significant valvular pathologies were excluded. Participants were asked to avoid moderate and intense physical activity as well as alcohol and coffee consumption in the 24 h preceding the study.

### Study design, setting, and instrumentation

**Fig. 1:**
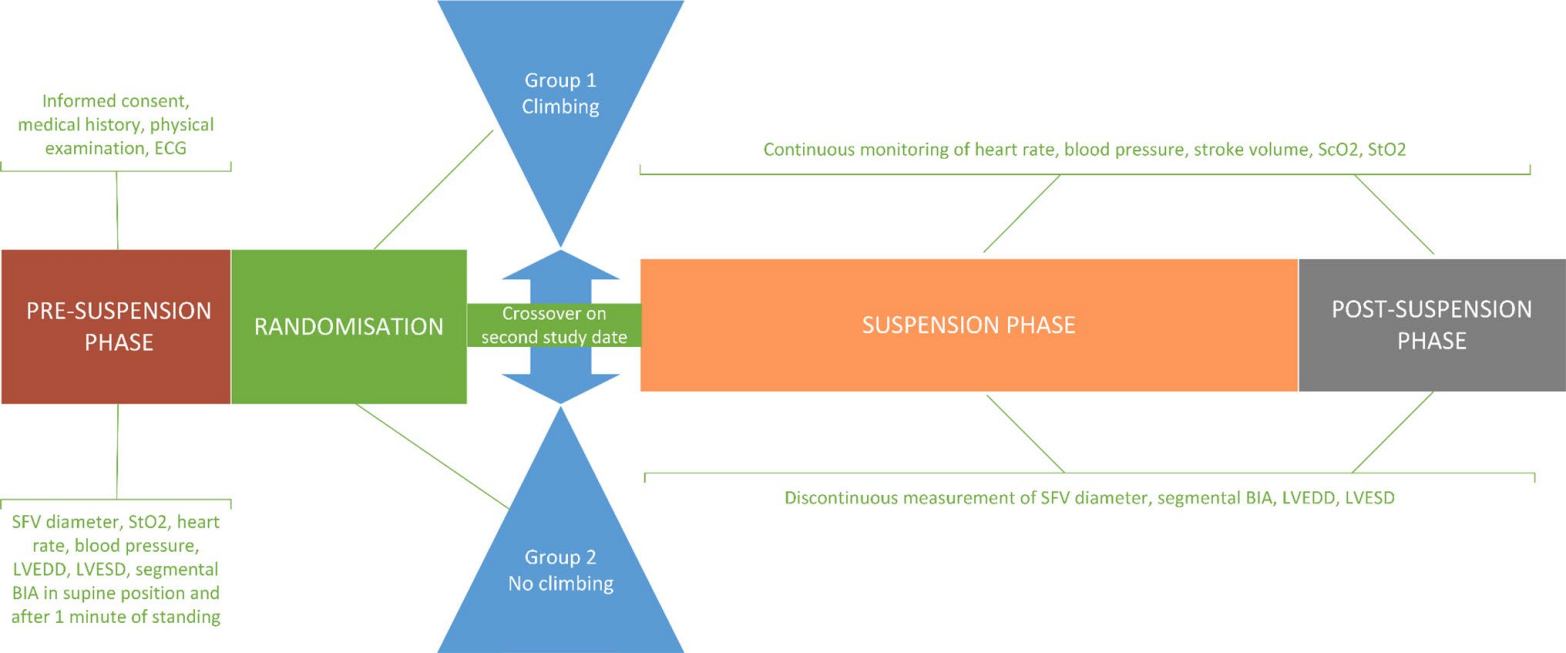
Study design. *ScO*_*2*_ cerebral oxygen saturation, *StO*_*2*_ tissue oxygen saturation of the calf muscles, *LVEDD* left ventricular end-diastolic diameter, *LVESD* left ventricular end-systolic diameter, *SFV* superficial femoral vein, *BIA* body impedance analysis

The study design is depicted in Fig. 1. The study was performed in an indoor climbing facility. In the “pre-suspension phase”, baseline values were obtained in a supine position (after 5 min of rest) and in a standing position (2 min after standing up) for the diameter of the right-sided superficial femoral vein (SFV), tissue oxygen saturation (StO_2_) of the right calf muscle, bioelectrical-impedance patterns of the lower leg (localized bioelectrical-impedance), heart rate (HR), blood pressure (BP), left ventricular end-systolic and end-diastolic diameter (LVESD and LVEDD) and cerebral oxygen saturation (ScO_2_). The diameter of the right SFV was measured with a linear, 5–10 MHz ultrasound probe (DP50, Mindray Bio-Medical Electronics Co., Ltd., Shenzhen, China). StO_2_ of the calf muscle was measured using near-infrared spectroscopy (NIRS) (O3™ Regional Oximetry, Masimo Corporation, Irvine, USA) with the probe placed posterolaterally at the level of maximal calf circumference. Single-frequency (50 kHz), tetrapolar, localized bioelectrical-impedance measurements were performed using the fibular head and the sinus tarsi as landmarks for the placement of the proximal and distal pair of electrodes, with the current driving and voltage sensing electrodes placed one next to the other (BIA 101 Anniversary device, Akern, Florence, Italy, phase sensitive BIA device). From phase sensitive bioelectrical-impedance analysis, resistance (*R*) and reactance (Xc) values can be derived. *R* is the opposition to the flow of an alternating current and is inversely related to fluid volume, whereas Xc indicates the capacitive component of the cell membranes (Lukaski 2013; Nescolarde et al. 2015). Thus, localized bioelectrical-impedance may be used to identify regional fluid accumulation and cell membrane integrity and function (Nescolarde et al. 2015). Additionally, localized bioelectrical-impedance has been related to blood circulation properties (Varlet-Marie et al. 2016). HR, BP and stroke volume (SV) were recorded non-invasively and continuously (beat-by-beat) (Nexfin^®^ HD, BMEYE B.V, Amsterdam, Netherlands) (Mehta and Arora 2014). From the inter-beat interval and the systolic BP recorded by the device, baroreceptor-sensitivity was calculated. The LVESD and LVEDD were determined in an apical four-chamber view using a 2.5–5 MHz probe (M5, Mindray Bio-Medical Electronics Co., Ltd., Shenzhen, China). ScO_2_ was measured using NIRS with the probe placed on the right-sided forehead.

Following baseline measurements, each participant was randomized into one of two groups: participants randomized into group 1 were asked to climb for 10 min at a moderate intensity before proceeding directly to the “suspension phase” without any discontinuity. Participants assigned to group 2 proceeded to the suspension phase without prior climbing. Adopting a crossover design, on a second study day, the participants switched the group (with a rest period of at least 48 h between the two tests). While climbing, participants were belayed in a sit harness according to the latest recommendations of the Austrian Alpine Club (Mössmer 2013).

During the “suspension phase”, the participants were freely suspended on a rope in a sit harness (Rock M, Salewa, Oberalp SPA, Bolzano, Italy, appropriate size selected according to manufacturer’s recommendation) about 50 cm above the ground. HR, BP and SV, as well as ScO_2_ and StO_2_ of the calf muscle were continuously monitored. The diameter of the SFV and the LVESD and LVEDD was determined every 2.5 min during the first 15 min of the suspension phase and in 5 min interval thereafter. Changes in localized bioelectrical-impedance were determined every 5 min. Clinical signs and symptoms were assessed continuously and dizziness, light-headedness, pallor, warmth, blurred vision and nausea were considered signs and symptoms of a pre-syncope. Participants were asked to report the maximum level of pain during the suspension phase on a 10-step numeric rating scale (NRS) (Breivik et al. 2008).

The suspension phase was interrupted if one of the interruption criteria listed in Table 1 was met.

**Table 1:** Criteria for interruption of the suspension phase

Participant wishes to interrupt the study
Predefined maximal duration of 60 min reached
Heart rate < 35 or > 160 bpm
Systolic blood pressure < 90 mmHg or > 200 mmHg
Glasgow coma scale ≤ 13
Decrease of cerebral oxygen saturation > 25% from baseline
Pre-syncopal signs and symptoms, i.e., dizziness, light-headedness, pale skin, a cold and clammy sweat, blurred vision and nausea

*Bpm* beats per minute

Participants were then immediately brought from the hanging position in a supine position (“post-suspension phase”). For another 15 min, the participants’ diameter of the SFV, StO_2_, localized bioelectrical impedance, HR, BP, SV, LVESD and LVEDD as well as ScO_2_ were monitored.

### Data analysis

Beat-to-beat data of HR, systolic BP and SV were first interpolated at 4 Hz (i.e., one value every 0.25 s was calculated) and then smoothed by means of a simple moving average of 30 s to reduce variability (for an example see Supplementary Figure 1). For the diameter of the SFV, StO_2_, HR, systolic BP, SV, LVEDD, LVESD and ScO_2_, a linear mixed model (LMM) was used to detect whether time, climbing, pre-syncope, pain (≤ 3 and > 3 on the NRS) and interactions of time with climbing, time with pre-syncope and time with pain had an effect during the suspension and post-suspension phases. For the LMM, different time points were defined: start of the suspension phase, 3 min before end of the suspension phase (when the most obvious changes of the parameters were noted), start of post-suspension phase and 5 min into the post-suspension phase. For SV, also 1 min before the end of the suspension phase was considered. Likewise, for the diameter of the SFV and StO_2_, an additional time point (minute 5 of the suspension phase) was used. The covariance structure for the residuals of the LMM was chosen by means of the Schwarz’s Bayesian Criterion (BIC) among diagonal, compound symmetry, unstructured and first-order auto-regression (AR(1)).

Baroreceptor-sensitivity was calculated as the ratio of the standard deviation of the inter-beat interval to the standard deviation of systolic BP, in accordance to previously proposed methods (Bernardi et al. 2010). Calculation was made every 5 s on a window size of 60 s. Baroreceptor-sensitivity was calculated for two time intervals: minutes 5–10 of the suspension phase (the first 5 min were not evaluated to have a more steady state) and the last minute of the suspension phase. The mean of the values of each interval was then calculated.

To compare two time points of a parameter as well as the means of the two intervals of the baroreceptor-sensitivity, the difference between them was used as dependent variable of a general linear model with participant as a random factor and climbing and pre-syncope as fixed factors. For multiple comparisons, the *P* values were then corrected by means of Holm–Bonferroni method; according to this method, as soon as a null hypothesis is not rejected, all subsequent null hypotheses in the procedure are considered as non-significant, and therefore their *P* value is denoted as *P* = n.s. in the text. In the climbing and non-climbing sessions, Student’s *t* test was used to compare mean values of a parameter. Test durations of participants with pre-syncope in both climbing and non-climbing sessions were compared by means of Wilcoxon signed-rank test.

SPSS version 24.0 (IBM Corp., Armonk, NY, USA) was used for the statistical analysis, while the interpolation and the smoothing of HR, systolic BP and SV as well as the calculations of the baroreceptor-sensitivity were realized in R version 3.4.1. (R Development Core Team 2015). Values are reported as mean ± SD and *P* < 0.05 was considered statistically significant. Unless stated otherwise, the mean values refer to the 40 test samples, i.e., with and without climbing prior to the suspension phase.

## Results

### Incidence, timing of pre-syncope, and the influence of climbing

**Fig. 2:**
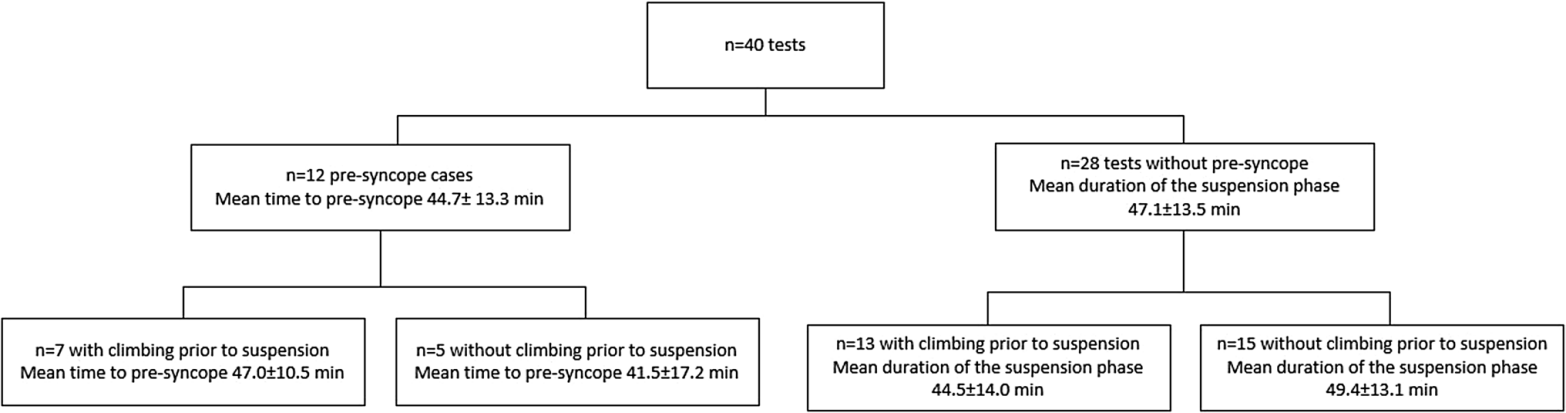
Cases with and without pre-syncope, divided in climbing and no-climbing prior to the suspension phase

Twelve (30%) out of the 40 tests were prematurely interrupted due to signs and symptoms of a pre-syncope, i.e., dizziness, light-headedness, pallor, warmth, blurred vision, and nausea (Fig. 2). The mean suspension time until the occurrence of the pre-syncope was 44.7 ± 13.3 min (minimum 13.4, maximum 59.7). Seven of the 12 (58.3%) pre-syncopal episodes occurred in participants who climbed before the suspension phase with a mean time to pre-syncope of 47.0 ± 10.5 min. The remaining five (41.7%) pre-syncopal episodes were observed without climbing prior to the suspension phase and occurred after a mean time of 41.5 ± 17.2 min. Four participants experienced pre-syncope during both tests (i.e., with and without prior climbing). The time until pre-syncope of these four participants was 40.4 ± 8.5 min with and 48.5 ± 8.1 min without climbing (*P* = 0.068).

The 28 tests without a pre-syncopal episode were interrupted because the predefined maximal duration of the suspension phase (60 min) was reached (43%) or due to pain (36%) or numbness/palsy (21%) in the lower extremities. The mean duration of the suspension phase in tests without a pre-syncope was 47.1 ± 13.5 min.

### Pain, numbness, and palsy in the lower extremities

During 24 tests (60%), participants reported pain in the lower extremities ≥ 3/10 on a 10-step NRS (Breivik et al. 2008). Among participants who experienced a pre-syncope, 75% (9 out of 12) reported a pain level ≥ 3 on the NRS. Although pain level did not differ between participants with and without a pre-syncope neither in the climbing session (5.1 ± 2.9 vs. 4.0 ± 2.8 on the NRS, *P* = 0.430) nor without previous climbing (3.5 ± 2.7 vs. 5.1 ± 2.9, *P* = 0.310), pre-syncope had an effect on pain level (*P* = 0.003, estimated marginal means for pain 2.9 without pre-syncope and 8.0 with pre-syncope) in a general linear model with climbing, pre-syncope and its interaction term as fixed factors and subject as random factor. Numbness was reported in 18 (45%) and palsy of the lower legs in 12 (30%) cases. Clinically, marked cyanosis of the lower legs was observed during the suspension phase.

### Results of the linear mixed models (LMM)

The analysis of the factors (i.e., time, climbing, pre-syncope, pain and interactions of time with climbing, time with pre-syncope and time with pain) that influenced the progresses of the diameter of the SFV, StO_2_, HR, systolic BP, SV, LVEDD, LVESD and ScO_2_ during the suspension and post-suspension phases is presented in Table 2.

**Table 2:** *P* values for fixed effects considered in the linear mixed model

Fixed effect	Dependent variable of linear mixed model^a^							
	HR	SBP	SV	StO_2_	ScO_2_	SFV	LVEDD	LVESD
Intercept	< 0.001	< 0.001	< 0.001	< 0.001	< 0.001	< 0.001	< 0.001	< 0.001
Time	< 0.001	< 0.001	< 0.001	< 0.001	0.004	< 0.001	0.001	0.047
Climbing	< 0.001	< 0.001	0.648	0.828	0.607	0.581	0.109	0.678
Pre-syncope	0.371	0.036	0.457	0.985	0.256	0.035	0.024	0.042
Pain	0.603	0.389	0.694	0.967	0.387	0.173	0.019	0.037
Climbing × time	< 0.001	0.034	0.535	0.043	0.052	0.708	0.475	0.947
Pre-syncope × time	0.007	< 0.001	0.111	0.487	0.071	0.224	0.548	0.202
Pain × time	0.256	0.250	0.696	0.943	0.884	0.250	0.379	0.214

*HR* heart rate, *SBP* systolic blood pressure, *SV* stroke volume, *StO*_*2*_ tissue oxygen saturation, *ScO*_*2*_ cerebral oxygen saturation, *SFV* diameter of the superficial femoral vein, *LVEDD* left ventricular end-diastolic diameter, *LVESD* left ventricular end-systolic diameter

^a^For all models the covariance structure for the residuals is AR(1), except for SBP and LVEDD compound symmetry

Time had an effect on all the parameters, while climbing (considering also its interaction with time) only on HR, systolic BP and StO_2_. For ScO_2_ the interaction of climbing with time did not reach statistical significance but was very close to it (*P* = 0.052). Interaction of pre-syncope with time had an effect on HR and SBP, indicating that participants with and without pre-syncope showed different progresses. Pre-syncope alone had an effect on SFV, LVEDD, and LVESD, indicating that participants with and without pre-syncope had a steady difference during the tests, i.e., participants who experienced a pre-syncope had a smaller diameter of the SFV (1.1 mm) and slightly larger ventricular diameters (1.2 mm for LVEDD and 1.9 mm for LVESD).

In the following paragraphs, for each parameter the results of the pairwise comparisons of the time points are described.

### Venous pooling

#### Diameter of the superficial femoral vein

**Fig. 3:**
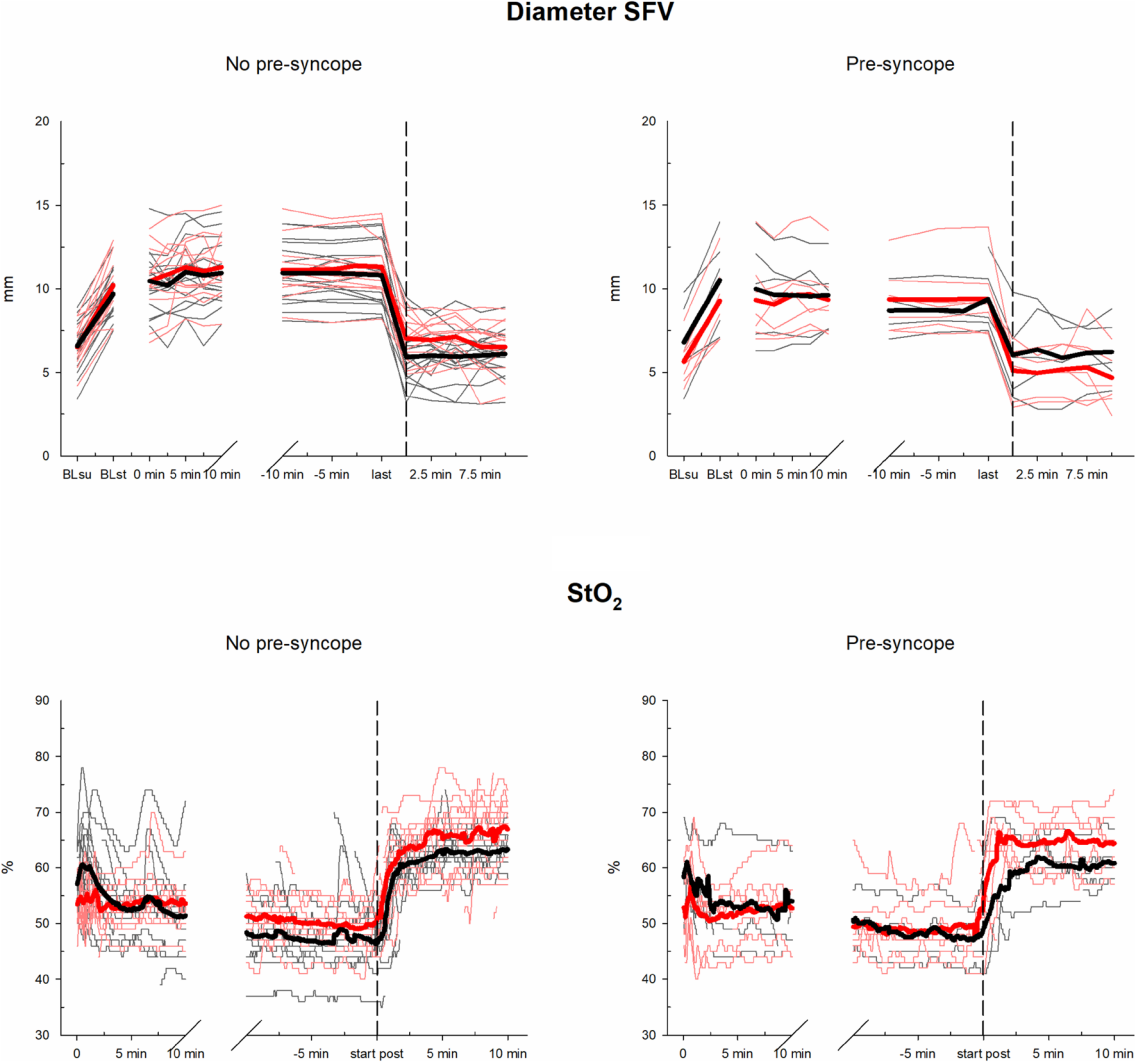
Progress of StO_2_ and SFV during suspension test. Black colour represents progresses without climbing before the test and red colour represents progresses with previous climbing. The two thicker lines represent the mean values of the two groups and the black vertical dashed line at the start of post-hanging phase. *BLsu* baseline value in supine position, *BLst* baseline value in standing position, *last* last measure before end of the test

The mean baseline diameter of the right SFV in the supine position was 6.4 ± 1.6 mm and increased to 9.9 ± 1.8 mm while standing (*P* < 0.001). The mean diameter of the SFV at the beginning of the suspension phase was 10.2 ± 2.1 mm. During the suspension phase, the diameter did not change significantly, neither in the pre-syncope cases nor in the cases without pre-syncope. Likewise, no influence of climbing on the SFV diameter was found. At 5 min of the post-suspension phase (in the supine position), a decrease in the diameter of the SFV was observed (10.6 ± 2 mm at the last measurement during suspension vs. 6.2 ± 1.6 mm at 5 min in the post-suspension phase, *P* < 0.001). SFV diameter changes during the tests are depicted in Fig. 3.

#### Tissue oxygen saturation (StO_2_) of the calf muscle

Baseline StO_2_ decreased from supine to the standing position (70.1 ± 6.6 vs. 56.4 ± 5.6%, *P* < 0.001). During the first 5 min of the suspension phase, a decrease in StO_2_ was detected in participants who did not climb (57.3 ± 5.5 vs. 52.4 ± 6.1%, *P* = 0.001), while StO_2_ did not change when participants had climbed before suspension (53.2 ± 4.4 vs. 52.8 ± 4.6%, *P* = 1.000). From minute 5 of the suspension phase to 3 min before end of the suspension phase, StO_2_ decreased further in both groups (52.6 ± 5.3 vs. 48.7 ± 5.6%, *P* = 0.002). During the first 5 min of the post-suspension phase, an increase in StO_2_ was observed (49.4 ± 6.5 vs. 63.6 ± 5.1%, *P* < 0.001). StO_2_ changes during the tests are depicted in Fig. 3.

#### Localized bioelectrical-impedance analysis

The resistance (*R*) (200.2 ± 18.6 vs. 193.6 ± 17.9, *P* < 0.001) and reactance (Xc) (27.7 ± 4.2 vs. 26.6 ± 3.7, *P* = 0.001) changed from supine to standing position. Xc further decreased from the start to the last measure during the suspension phase (26.4 ± 3.6 vs. 22.9 ± 3.1, *P* < 0.001) while *R* (192.3 ± 18.3 vs. 190.7 ± 17.6, *P* = 0.201) did not change. The general linear model showed no effect of climbing (*P* = 0.514 and *P* = 0.253 for *R* and Xc, respectively) or pre-syncope (*P* = 0.981 and *P* = 0.701 for *R* and Xc, respectively) from the start to the last measure during the suspension phase.

### Hemodynamic changes

#### Heart rate

**Fig. 4:**
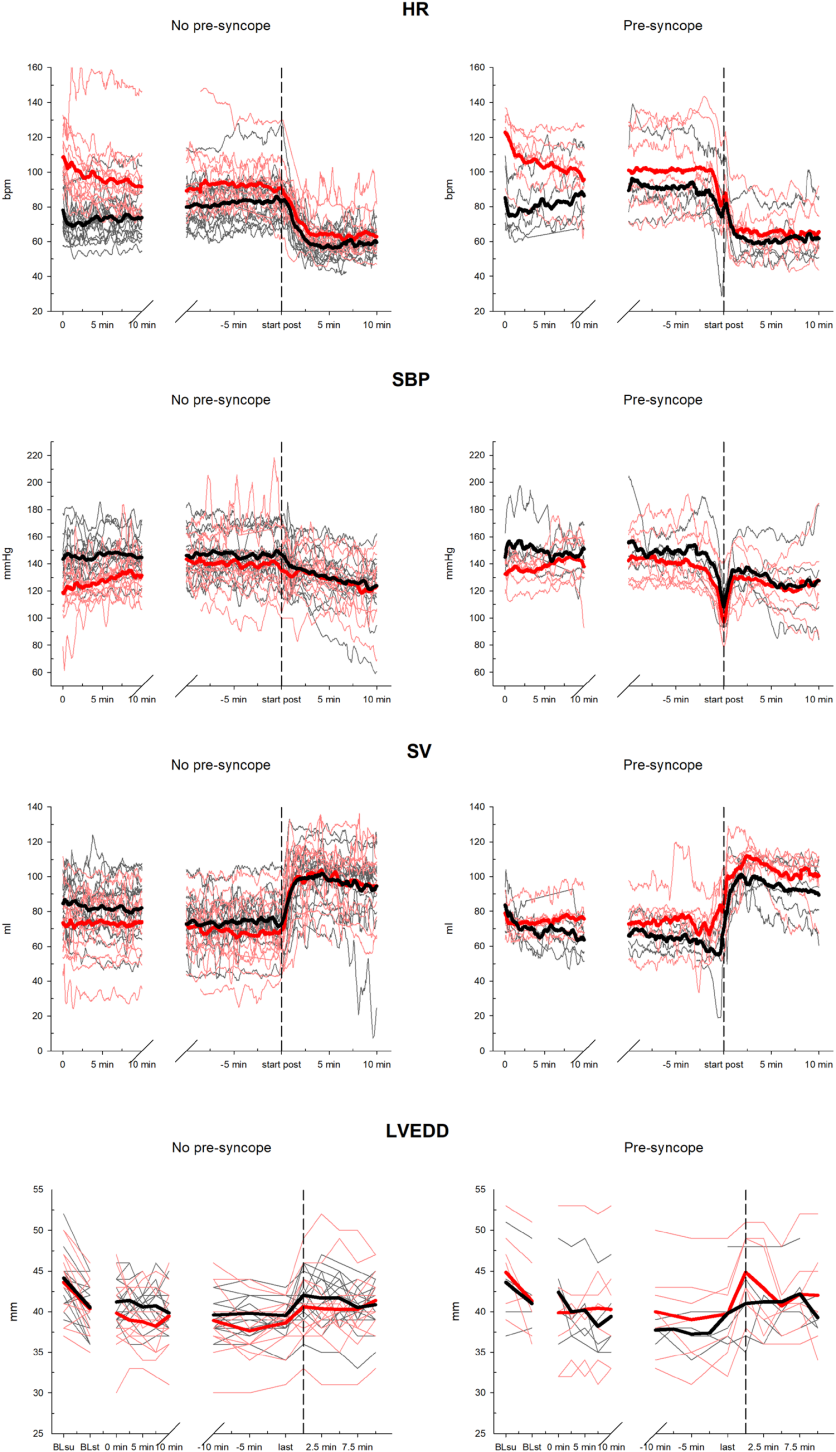
Progress of HR, SBP, SV and LVD during suspension test. Black colour represents progresses without climbing before the test and red colour represents progresses with previous climbing. The two thicker lines represent the mean values of the two groups and the black vertical dashed line at the start of post-hanging phase. *BLsu* baseline value in supine position, *BLst* baseline value in standing position, *last* last measure before end of the test

Baseline HR increased from supine to the standing position (63.1 ± 7.9 vs. 72.4 ± 9.4 bpm, *P* < 0.001). During suspension, HR decreased from the start to 3 min before the end of the suspension phase in participants who had climbed before (113.3 ± 15.9 vs. 96.0 ± 18.5 bpm, *P* < 0.001) but did not change in participants who had not climbed (79.8 ± 14.4 vs. 84.4 ± 15.2 bpm, respectively, *P* = 0.098). In participants with pre-syncope, HR decreased in the last 3 min before the pre-syncopal event (96.3 ± 22.4 vs. 82.2 ± 19.4 bpm, *P* = 0.043) and decreased further during the first 5 min of the post-suspension phase (82.2 ± 19.4 vs. 62.9 ± 13.5 bpm, *P* = 0.033). In participants who did not manifest a pre-syncope, HR remained stable until the end of the suspension phase and decreased only in the first 5 min of the post-suspension phase (86.4 ± 15.8 vs. 60.3 ± 10.1 bpm, *P* < 0.001). HR changes are depicted in Fig. 4.

#### Systolic blood pressure

Systolic BP increased at baseline from supine to standing (121.1 ± 13.2 vs. 138.7 ± 14.7 mmHg, *P* < 0.001). In participants who had not climbed before the test, systolic BP did not change from the start of the suspension phase until 3 min before end of the suspension phase (143.8 ± 12.8 vs. 148.0 ± 15.3 mmHg, *P* = 0.272) but increased in participants who had climbed before (123.6 ± 17.1 vs. 139.2 ± 21.8 mmHg, *P* = 0.034). In participants with pre-syncope, systolic BP decreased significantly in the last 3 min before the pre-syncopal event (142.5 ± 18.6 vs. 101.6 ± 17.4 mmHg, *P* = 0.001) while it did not change in participants without a pre-syncope (144.1 ± 19.6 vs. 141.7 ± 20.4 mmHg, *P* = n.s.). During the first 5 min of the post-suspension phase, systolic BP decreased in participants without a pre-syncope (141.7 ± 20.4 vs. 128.9 ± 17.8 mmHg, *P* = 0.038) and increased in participants who had a pre-syncope (though not reaching statistical significance; 101.6 ± 17.4 vs. 124.9 ± 18.2 mmHg, *P* = n.s.). Systolic BP changes are depicted in Fig. 4.

#### Stroke volume

In both participants with and without climbing before the suspension phase, stroke volume decreased from the beginning of the suspension phase to 3 min before the end of the suspension (80.1 ± 14.7 vs. 69.7 ± 15.9 ml, *P* = 0.007). No statistically significant reduction of stroke volume in participants with a pre-syncope was detected before the occurrence of signs and symptoms of pre-syncope. In the first 5 min of the post-suspension phase, SV increased (73.5 ± 16.5 vs. 98.9 ± 14.9 ml, *P* < 0.001). SV changes are depicted in Fig. 4.

#### Left ventricular diameters

##### LVEDD

Baseline LVEDD decreased from supine to standing (44.0 ± 4.2 vs. 40.7 ± 3.8 mm, *P* < 0.001). During the suspension phase, no significant change in LVEDD was observed. LVEDD increased from the last measure taken before the end of suspension to the start of post-suspension phase (39.3 ± 3.6 mm vs. 41.9 ± 4.2 mm, *P* = 0.001). LVEDD changes are depicted in Fig. 4.

##### LVESD

LVESD did not change at baseline from supine to standing (27.6 ± 3.9 mm vs. 26.5 ± 3.6 mm, *P* = 0.098). No pairwise comparison of the time points was significant.

### Cerebral oxygen saturation (ScO_2_)

ScO_2_ did not change from supine to standing at baseline (67.9 ± 4.7 vs. 67.2 ± 4.3%, *P* = 0.199). Between the start and 3 min before the end of suspension phase, a statistically significant increase in ScO_2_ was observed in participants who did not climb before suspension (66.3 ± 4.5 vs. 69.9 ± 5.6%, *P* = 0.001; relative change from start + 5.4% ± 5.2%), whereas in those with prior climbing, ScO_2_ remained constant (70.3 ± 5.8 vs. 69.8 ± 5.9%, *P* = n.s.; relative change from start − 0.5% ± 7.1%). In the participants with pre-syncope, ScO_2_ did not change significantly in the 3 min preceding the pre-syncope (73.0 ± 6.2 vs. 69.1 ± 6.2%, *P* = n.s.; relative changes from start + 3.8% ± 8.6% vs. − 1.8% ± 7.1%). Also during the first 5 min of the post-suspension phase, ScO_2_ did not change (68.4 ± 5.5 vs. 69.5 ± 5.2%, *P* = 0.194; relative changes from start + 0.4% ± 6.8% vs. + 2.0% ± 7.1%).

### Baroreceptor-sensitivity

39 out of 40 tests were included in the baroreceptor-sensitivity analysis (one with missing data was excluded). Overall, no time effect of pre-syncope on baroreceptor-sensitivity was found (*P* = 0.308). Nonetheless, in the first minutes during suspension in the tests without prior climbing, baroreceptor-sensitivity was higher in participants without syncope compared to the ones with pre-syncope (12.6 ± 3.6 vs. 7.7 ± 3.8, *P* = 0.026). In the tests with prior climbing, no such differences were found (7.2 ± 3.3 vs. 6.7 ± 3.5, *P* = 0.782).

## Discussion

In our study, we observed a pre-syncope during suspension in a sit harness in 30% of all tests. Time to pre-syncope was highly variable and unpredictable with a sudden occurrence of signs and symptoms without a prodromal stage. Venous pooling in the lower leg could be evidenced very early in all tests; however, the reduced preload did not seem to have any relevant impact on macro-hemodynamics. In participants with a pre-syncope, a sudden decrease in HR and systolic BP was observed, resembling a vagal mechanism.

The principal aim of our study was to clarify the pathophysiology of suspension syndrome and particularly to differentiate between an important reduction in cardiac preload due to venous pooling, and a vagally mediated circulatory collapse. A hemodynamically relevant venous pooling, causing a reduction in cardiac preload, would be expected to elicit compensatory mechanisms to preserve cardiac output, such as progressive tachycardia. Moreover, a decrease in LVEDD and LVESD and finally a drop of BP would be anticipated (Pacagnella et al. 2013). In contrast, a vagal event would be associated with vasodepressor hypotension and/or inappropriate bradycardia (Kapoor 2000; Shen et al. 2017).

To measure venous pooling in the lower leg, we used the diameter of the SFV, the StO_2_ of the calf muscle and the localized bioelectrical-impedance. A shift of blood to dependent parts of the body leads to filling of capacitance vessels in the leg with an increase in diameter (Marshall et al. 2014). Using ultrasound, the increase in diameter of large veins, such as the SFV, can easily be monitored over time. However, measurement of the orthostatic blood volume accumulation in the small vessels is more challenging. To track intravascular orthostatic volume changes within the small blood vessels of the lower leg, a new four-wavelength generation NIRS monitor has been used (Ferraris et al. 2018). NIRS tissue oxygen saturation measurement is made on a mixture of both venous and arterial blood with a mean ratio of this mixture estimated to be 70% venous–30% arterial (Benni et al. 2018). A decrease in StO_2_ thus indicates either accumulation of de-saturated venous blood or a reduced oxygen delivery (Bartok and Schoeller 2004; Binzoni et al. 2000, 2003; Habazettl et al. 2016; Hachiya et al. 2004; Truijen et al. 2012). The observed decrease in StO_2_ during suspension was attributed to an accumulation of venous blood rather than a reduced oxygen supply, as no compression or flow limitation of the arterial vessels (e.g., by the harness straps) was observed with ultrasound and stroke volume and arterial oxygen saturation were constant. As a third method to monitor regional circulation and fluid accumulation in the calf muscle-localized bioelectrical-impedance components have been utilized (Lukaski 2013). Our study is the first to quantify venous pooling during suspension using these three distinct modalities. We found that immediately after the beginning of the suspension phase, a marked and rapid accumulation of venous blood in the superficial femoral vein occurred. That is, the diameter of the SFV immediately increased to values comparable to those measured at baseline after 2 min of upright standing. However, during the suspension phase, the diameter of the SFV did not progressively increase, supposedly because the maximum distending capacity of the venous vessel had been already reached. Concomitantly to the initial increase in SFV diameter, a remarkable decrease in StO_2_ measured with NIRS was observed. Most likely, this can be explained by an accumulation of desaturated blood in venules (Habazettl et al. 2016), which was in line with the observation of a marked cyanosis of the feet, observed in nearly all participants. After the initial pronounced drop of the StO_2_, a steady but more gradual decrease was observed. Together with the constant diameter of the SFV, this finding indicates progressive venous pooling at the level of small venous vessels. Similar to the SFV diameter, *R* values during suspension were comparable to the values attained at baseline during standing and were lower than the values recorded in the supine position, which might be a consequence of a gravitationally induced blood pooling in abdomen and legs. Interestingly, whereas *R* remained stable in the course of the suspension, which indicates no additional fluid volume increase, Xc further decreased. This might indicate fluid shifts between extracellular and intracellular compartments (Gatterer et al. 2014) and/or changes in blood circulation properties (Varlet-Marie et al. 2016), which could have occurred in the course of the suspension. Yet, the exact meaning of the changed Xc cannot be explained by this study and at present this interpretation has to be viewed with caution.

The accumulation of blood in the dependent body regions, however, seemed not to have any substantial impact on macro-hemodynamics, which we assessed by continuous measurement of HR, BP and SV and intermittent quantification of LVEDD and LVESD. We found that, despite venous pooling, only SV slightly decreased during suspension. Despite the reduction in SV, blood pressure remained constant because of a compensatory increase in vascular resistance. Importantly, venous pooling did not elicit a compensatory increase in HR, which would definitely be expected if the preload was reduced to a significant degree, similarly as seen in hypovolemic/hemorrhagic shock (Salomao et al. 2015). Furthermore, LVEDD did not decrease, and, taken together, our findings contradict the hypothesis of a marked preload reduction leading to an “empty beating heart”. Quite in contrast, all pre-syncope cases in our study followed a uniform pattern, favoring the hypothesis of a vagal event. Indeed, the pre-syncope was characterized by a pronounced decrease in systolic BP and HR as in a vasovagal syncope with a mixed vasodepressive and cardioinhibitory pattern. The haemodynamic findings were preceded by a sudden occurrence of dizziness, light-headedness, pale skin, warmth, blurred vision or nausea, lasting from a few seconds to a few minutes. The haemodynamic pattern observed in our study is identical to that found in patients with a vasovagal syncope in the tilt table test (Brignole et al. 2000; Gaggioli et al. 2000). Thus, we hypothesize that the susceptibility of an individual to develop suspension syndrome could be predicted in a tilt test. However, this needs to be proven in a future trial.

The mechanism behind a vagal syncope has not been totally elucidated (Mosqueda-Garcia et al. 2000). One hypothesis implies the ventricular muscle to contract on a nearly empty chamber, which, in turn, is thought to stimulate afferent signals from the left ventricle to the brainstem, inhibiting sympathetic outflow to blood vessels and the heart, resulting in arterial hypotension and bradycardia (Bezold-Jarisch reflex) (Mark 1983). Our findings do not corroborate this theory, as referred to above. Another hypothesis involves a dysfunction of the arterial baroreceptor reflex. The baroreceptor reflex is a primary homeostatic mechanism that maintains arterial blood pressure within narrow limits via changes in cardiac output and systemic vascular resistance, mediated by the autonomic nervous system. Several authors have advocated defective baroreflex function as a potential mechanism accounting for the development of vasovagal syncope (Ellenbogen et al. 1997; Mosqueda-Garcia et al. 1997). In our study, participants experiencing a pre-syncope showed lower baroreceptor-sensitivity values during the initial phase of suspension (only visible in the trial without prior climbing), though, baroreceptor-sensitivity was unchanged during suspension. Particularly, no change in baroreceptor-sensitivity was found in the minutes preceding the pre-syncope. This neither confirms nor refutes the hypothesis of a baroreflex dysfunction in the pathophysiology of vagal syncope.

Pain is a known trigger for vagally mediated syncopal events (Hainsworth 2003) and in our study, 75% of participants who experienced a pre-syncope reported significant pain (≥ 3 on a NRS). By means of a general linear model, we found that pain level differed between participants with and without pre-syncope, thus we can conclude that pain seems to be an important factor contributing to the vagal event.

The time to pre-syncope was variable and unpredictable and the signs and symptoms of pre-syncope occurred suddenly without a prodromal stage. The shortest time to pre-syncope in our study was 13.4 min with a mean time to pre-syncope of 44.7 min and a maximal time of 59 min, respectively. This is comparable to a recent study on suspension syndrome, which found a mean time to syncope of 30 min with a range from 20 to 36 min (Lanfranconi et al. 2017). Four volunteers from our study experienced a pre-syncope during both tests, with and without prior climbing, and two of them had a history of a past vagal syncope. This supports the hypothesis that, though the vagal response is present in all healthy humans, inter-individual differences in susceptibility seem to exist (Alboni et al. 2007) and persons prone to vagally mediated syncopal events might be more susceptible to suspension syndrome.

Similarly to a post-exercise syncope (Lowe and Petch 2000; O’Connor et al. 1999; Shen et al. 2017), we assumed that climbing before suspension would increase the susceptibility and decrease the time until the occurrence of suspension syndrome, particularly if a vagal mechanism was involved. In the four participants who experienced a pre-syncope in both tests, the mean time to pre-syncope was shorter when they climbed before suspension and of the remaining four pre-syncopal cases, three occurred after climbing, supporting our hypothesis.

**Fig. 5:**
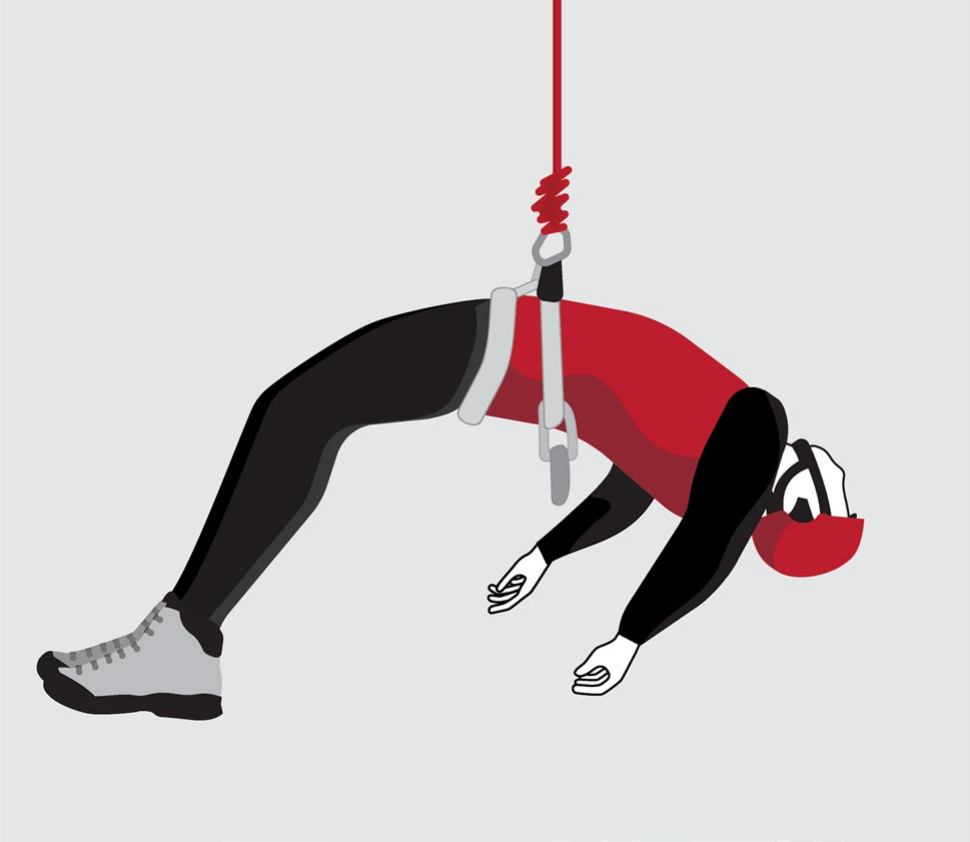
In an unconscious person suspended in a sit harness, the attachment point of the rope, located at the level of the hips will represent the highest point

We showed that time until loss of consciousness while suspended on a rope is unpredictable. It is therefore recommended, that persons suspended on a rope and not able to free themselves should be rescued as soon as possible, not least because loss of consciousness while hanging on a rope can rapidly become life threatening: While the syncopal fall normally will accomplish a horizontal position, allowing a rapid restoration of cerebral perfusion and rapid recovery (Weimer and Zadeh 2009), this is not the case when hanging vertically on a rope. In an unconscious person suspended in a sit harness, the attachment point of the rope, located at the level of the hips will represent the highest point (Fig. 5), impeding the return flow of blood accumulated in the veins of the lower legs. Thus, perfusion of the vital organs may not be restored and the person may eventually die.

Until today, it is unclear how to position a casualty appropriately once rescued from suspension. Although most authors recommend a supine position (Adisesh et al. 2011; Thomassen et al. 2009) according to the advanced life support guidelines (Soar et al. 2015), some still advise a semi-recumbent position first, with the idea to avoid an acute ventricular volume overload. In our study, we decided to bring participants with a pre-syncope in a supine position. As expected, LVEDD increased in the horizontal position, however, the diameter did not even reach the supine baseline value, contradicting the hypothesis of a ventricular overdistension. Moreover, all participants with a pre-syncope recovered within seconds in the supine position. Therefore, we recommend that persons hanging on a rope and showing signs of a suspension syndrome should be rescued and put into supine position as soon as possible.

## Limitations

Participants were asked to move their legs as little as possible while suspended, to facilitate the measurements of venous pooling (particularly ultrasonografic measurements). The reduced leg movements might have influenced the degree of venous pooling and the time to pre-syncope. In real life, persons hanging freely on a rope will probably move their legs spontaneously, possibly reducing venous pooling, and delaying loss of consciousness. However, syncope will remain a sudden event and exhaustion, injuries, hypoglycemia, hypothermia, pain etc. might preclude antigravity muscle activity when being suspended on a rope outdoors.

We used echocardiography to monitor left ventricular dimensions as a measure of ventricular filling. Ventricular diameter is not an ideal parameter for preload and only a considerable ventricular underfilling could have been detected. Moreover, assessment of right ventricular dimensions might have been more appropriate to exclude ventricular volume overload once the participants have been brought in a supine position. However, especially during the suspension phase, the acoustic window was not ideal to depict the right side of the heart. Though the dimensions of the right ventricle were not assessed directly, no other signs of right ventricular volume overload, such as a diastolic septal displacement, were apparent. Even though localized bioimpedance analysis has been shown to identify local fluid accumulation (Nescolarde et al. 2015) and to be related to blood rheology (Varlet-Marie et al. 2016), the method is not well validated yet and thus these results have to be interpreted with caution.

## Conclusions

We found a surprisingly high rate (30%) of pre-syncopal episodes in our study and in all cases, a uniform circulatory and hemodynamic pattern could be observed: Blood pools in the veins of the lower legs, however, no relevant impact on macro-hemodynamics was detected. Pre-syncope was characterized by a sudden decrease in BP and HR, indicating a vagally driven mechanism with a mixed vasodepressive and cardioinhibitory pattern; pain during suspension may act as a trigger to evoke the reflex. Time to pre-syncope is unpredictable and persons suspended on a rope should be rescued and put into a supine position as soon as possible.

## Electronic supplementary material

Below is the link to the electronic supplementary material.

Supplementary material 1 (TIF 674 KB)

## Abbreviations

BIA: Body impedance analysis
BP: Blood pressure
HR: Heart rate
LMM: Linear mixed model
LVEDD: Left ventricular end-diastolic diameter
LVESD: Left ventricular end-systolic diameter
*H*: Height
NIRS: Near-infrared spectroscopy
*R*: Resistance
SBP: Systolic blood pressure
SFV: Superficial femoral vein
SV: Stroke volume
ScO_2_: Cerebral oxygen saturation
StO_2_: Tissue oxygen saturation
Xc: Reactance
