# Correction to: Suspension syndrome: a potentially fatal vagally mediated circulatory collapse—an experimental randomized crossover trial

**DOI:** 10.1007/s00421-019-04141-6

**Published:** 2019-04-19

**Authors:** Simon Rauch, K. Schenk, G. Strapazzon, T. Dal Cappello, H. Gatterer, M. Palma, M. Erckert, L. Oberhuber, B. Bliemsrieder, H. Brugger, P. Paal

**Affiliations:** 1 Institute of Mountain Emergency Medicine, Eurac Research, Viale Druso 1, 39100 Bolzano, Italy; 2 Department of Sports Science, Medical Section, University of Innsbruck, 6020 Innsbruck, Austria; 3 Department of Anesthesiology, University Hospital LMU Munich, 80337 Munich, Germany; 4 Department of Sports Medicine, Pro Motus, 39100 Bolzano, Italy; 5 Department of Cardiology, F. Tappeiner Hospital, 39012 Merano, Italy; 6 Department of Internal Medicine I, Gastroenterology, Hepatology, Metabolism and Endocrinology, Medical University Innsbruck, 6020 Innsbruck, Austria; 7 Department of Anesthesiology, Garmisch-Partenkirchen Medical Center, 82467 Garmisch-Partenkirchen, Germany; 8 Department of Anesthesiology and Intensive Care Medicine, Brothers of St. John of God Hospital, Paracelsus Medical University, Kajetanerplatz 1, 5010 Salzburg, Austria

## Correction to: European Journal of Applied Physiology 10.1007/s00421-019-04126-5

The original version of this article unfortunately contained a mistake. Information was missing in the acknowledgements section. The correct information is given below.

**Acknowledgements** We thank the Mountain Rescue South Tyrol (Bergrettungsdienst im Alpenverein Südtirol) for putting their indoor climbing facility at our disposal. Furthermore, we thank the companies Mindray Bio-Medical Electronics Co., Ltd., Masimo Corporation, Akern and Salewa for lending the equipment. We would like to acknowledge the support of Prof. Luciano Bernardi in interpreting the data on baroreflex sensitivity. The authors thank the Department of Innovation, Research and University of the Autonomous Province of Bozen/Bolzano for covering the Open Access publication costs.

