# Supplementary figures and images for: Suspension syndrome: a potentially fatal vagally mediated circulatory collapse—an experimental randomized crossover trial
Source: Eur J Appl Physiol. 2019 Mar 20;119(6):1353–65. doi: 10.1007/s00421-019-04126-5 (PMC6517360; doi:10.1007/s00421-019-04126-5)

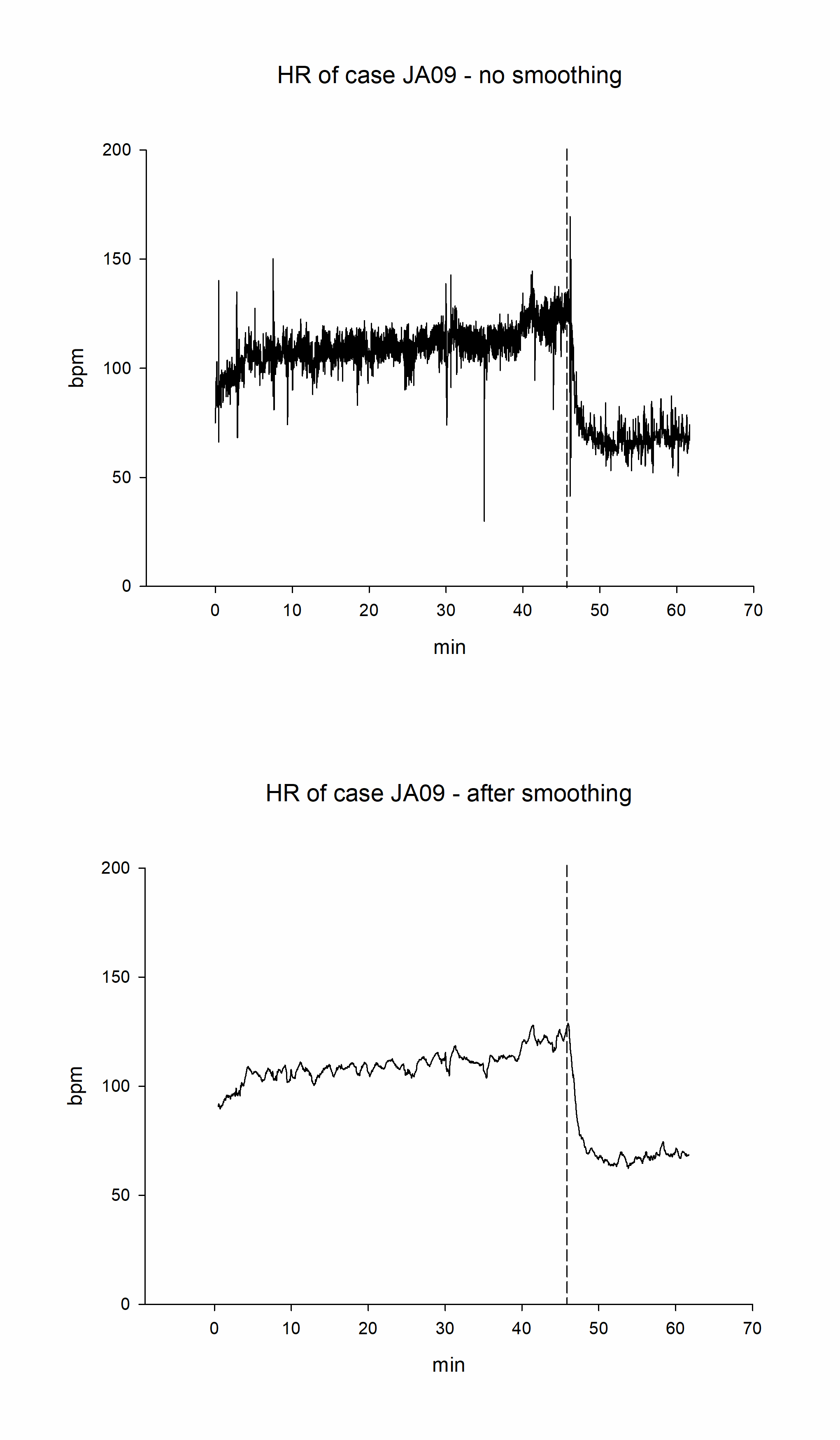

Supplement: Supplementary file 1 — Supplementary material 1 (TIF 674 KB) [file 421_2019_4126_MOESM1_ESM.tif]
